# Supplementary material for: Nickel mine soil is a potential source for soybean plant growth promoting and heavy metal tolerant rhizobia
Source: PeerJ. 2022 Apr 21;10:e13215. doi: 10.7717/peerj.13215 (PMC9035279; doi:10.7717/peerj.13215)
Supplement: Table S1 — Note: The differences in MIC and MLC values were not tested due to their zero variance. [file peerj-10-13215-s001.docx]

| Isolate | Cd^2+^ | | Cr^6+^ | | Cu^2+^ | | Ni^2+^ | | Zn^2+^ | |
| --- | --- | --- | --- | --- | --- | --- | --- | --- | --- | --- |
|  | MIC | MLC | MIC | MLC | MIC | MLC | MIC | MLC | MIC | MLC |
| **YN5** | 8 | 100 | 4 | 16 | 16 | 60 | 12 | 40 | 220 | 300 |
| **YN8** | 4 | 4 | 4 | 16 | 4 | 20 | 8 | 12 | 4 | 4 |
| **YN10** | 4 | 4 | 8 | 16 | 12 | 40 | 12 | 12 | 4 | 16 |
| **YN11** | 8 | 100 | 8 | 16 | 12 | 60 | 4 | 40 | 4 | 80 |
