# Supplementary material for: Nickel mine soil is a potential source for soybean plant growth promoting and heavy metal tolerant rhizobia
Source: PeerJ. 2022 Apr 21;10:e13215. doi: 10.7717/peerj.13215 (PMC9035279; doi:10.7717/peerj.13215)
Supplement: Table S2 [file peerj-10-13215-s002.docx]

Table S2. Primer sequences used in this study.

| **Target gene** | **Primer** | **sequence (direction 5’-3’)** |
| --- | --- | --- |
| 16S rRNA | 27F | GAGTTTGATCACTGGCTCAG |
|  | 1492R | TACGGCTACCTTGTTACGACTT |
| *atpD* | atpD255F | GCTSGGCCGCATCMTSAACGTC |
|  | atpD782R | GCCGACACTTCMGAACCNGCCTG |
| *recA* | recA63F | ATCGAGCGGTCGTTCGGCAAGGG |
|  | recA555R | CGRATCTGGTTGATGAAGATCACCAT |
| *glnII* | glnII12F | YAAGCTCGAGTACATYTGGCT |
|  | glnIItsR | SGAGCCGTTCCAGTGGTGTCG |
| *rpoB* | rpoB454F | ATCGTCTCGCAGATGCACCG |
|  | rpoB1364R | TCGATGTCGTCGATYTCGCC |
| *nifH* | nifHF | TACGGNAARGGSGGNATCGGCAA |
|  | nifHI | AGCATGTCYTCSAGYTCNTCCA |
