# Supplementary material for: Nickel mine soil is a potential source for soybean plant growth promoting and heavy metal tolerant rhizobia
Source: PeerJ. 2022 Apr 21;10:e13215. doi: 10.7717/peerj.13215 (PMC9035279; doi:10.7717/peerj.13215)
Supplement: Table S3 [file peerj-10-13215-s003.docx]

**Table S3**. Reference strains used in 16S rRNA phylogenetic tree.

| **16S rRNA** | | | **16S rRNA** | | |
| --- | --- | --- | --- | --- | --- |
| Strain | ID | Country | Strain | ID | Country |
| CFNEI 156 | NR025251 | Mexico | LMG 6133 | NR118988 | UK |
| CCBAU110 | NR114561 | China | ITTG-R7 | NR115768 | Mexico |
| USDA 205 | NR112784 | Japan | ITTG S70 | NR116265 | Mexico |
| CCBAU 251167 | NR149806 | China | ICMP 13649 | MT759986 | China |
| LMG 7837 | NR026096 | UK | ATCC 33212 | NR114539 | USA |
| CCBAU 23380 | NR151923 | China | CCBAU 65729 | NR133053 | China |
| CCBAU 05684 | NR117540 | China | Ch11 | NR118559 | New Zealand |
| NBRC 100382 | NR113889 | Japan | 20SJ14W-33 | MW010434 | Korea |
| CCBAU 65732 | NR133052 | China | KFB 330 | KP172482 | Serbia |
| ORS 1400 | NR115250 | France | 39/7 | NR117203 | Poland |
| LMG 14919 | NR114988 | Belgium | NRCPB10 | NR116874 | India |
| YIC 4027 | NR153737 | China | YIC 5082 | NR157010 | China |
| ORS 1407 | NR115249 | France | M15 | MH569334 | China |
| CCBAU 71714 | NR114614 | China | ICMP 5856 | MT759983 | China |
