# Supplementary material for: Nickel mine soil is a potential source for soybean plant growth promoting and heavy metal tolerant rhizobia
Source: PeerJ. 2022 Apr 21;10:e13215. doi: 10.7717/peerj.13215 (PMC9035279; doi:10.7717/peerj.13215)
Supplement: Table S4 [file peerj-10-13215-s004.docx]

**Table** **S4**. Reference strains used in housekeeping genes-MLSA phylogenetic tree.

|  | ***atpD*** | | | ***recA*** | | | |
| --- | --- | --- | --- | --- | --- | --- | --- |
| **No.** | Strain | ID | Country | Strain | ID | Country | |
| 1 | YIC 5082 | KP142172 | China | YIC 5082 | KP142170 | China | |
| 2 | YIC4121 | KR154039 | China | YIC4121 | KR154009 | China | |
| 3 | NCPPB 2437 | AJ294407 | UK | NCPPB 2437 | AB253188 | Japan | |
| 4 | HAMBI 1812 | KF206562 | Finland | HAMBI 1812 | KF206814 | Finland | |
| 5 | CCBAU 101002 | JQ308329 | China | CCBAU 101002 | JQ308335 | China | |
| 6 | LMG 18254 | AM418782 | Belgium | LMG18254 | AB253205 | Japan | |
| 7 | HAMBI 1816 | KF206564 | Finland | HAMBI 1816 | KF206819 | Finland | |
| 8 | WYCCWR 11279 | MH778818 | China | WYCCWR 11279 | MH778848 | China | |
| 9 | CCBAU 33202 | EF579929 | China | CCBAU 33202 | EF579941 | China | |
| 10 | LMG 14919 | AM418767 | Belgium | HAMBI 1552 | DQ411946 | Mexico | |
| 11 | LMG 19920 | AM418754 | Belgium | LMG 19920 | AM182135 | Belgium | |
| 12 | CCBAU 05684 | GU994042 | China | CCBAU 05684 | GU994055 | China | |
| 13 | LMG 7837 | AM418756 | Belgium | LMG 7837 | AM182138 | Belgium | |
| 14 | CCBAU 23380 | KJ556470 | China | CCBAU 23380 | KJ556427 | China | |
| 15 | LMG 17930 | AM418745 | Belgium | LMG 17930 | AM182148 | Belgium | |
| 16 | HAMBI 2910 | GU994045 | China | ITTG-R7 | DQ411951 | Mexico | |
| 17 | HAMBI 220 | AJ294403 | UK | HAMBI 220 | AJ294383 | UK | |
| 18 | ENCBTM 31 | JN009840 | Mexico | CFNEI 156 | DQ411943 | Mexico | |
| 19 | LMG 19227 | AM418771 | Belgium | HAMBI 1489 | DQ411947 | Mexico | |
| 20 | USDA 205 | AJ294402 | UK | USDA 205 | AJ294379 | UK | |
| 21 | USDA 1002 | AJ294400 | UK | USDA 100 | AJ294382 | UK | |
|  | ***glnII*** | | | ***rpoB*** | | | |
| **No.** | Strain | ID | Country | Strain | ID | | Country |
| 1 | YIC 5082 | KP202170 | China | YIC 5082 | NZMRDH0100004 | | China |
| 2 | YIC4121 | KR154024 | China | YIC4121 | MT028484 | | China |
| 3 | NCPPB 2437 | JN580714 | Finland | NCPPB 2437 | JN589757 | | Japan |
| 4 | NBRC 13261 | BBJU01000003 | Japan | HAMBI 1812 | KF206905 | | Finland |
| 5 | CCBAU 101002 | JQ308332 | China | CCBAU 101002 | KM491524 | | Finland |
| 6 | HAMBI 2409 | KF206740 | Finland | LMG 18254 | EF217309 | | UK |
| 7 | HAMBI 2546 | KF206753 | Finland | HAMBI 503 | KF206807 | | Finland |
| 8 | WYCCWR 11279 | MT169727 | China | WYCCWR 11317 | NZJACGBJ010000033 | | China |
| 9 | CCBAU | EF579935 | China | CCBAU 33202 | FJ392877 | | China |
| 10 | HAMBI 1552 | HM997093 | China | LMG 14919 | AM295380 | | Belgium |
| 11 | LMG 19920 | KR818746 | Spain | LMG 19920 | AM295387 | | Belgium |
| 12 | CCBAU 05684 | GU994060 | China | CCBAU 05684 | GU994049 | | China |
| 13 | ORS609 | AF169589 | UK | LMG 7837 | AM295362 | | Belgium |
| 14 | CCBAU 05593 | HQ174492 | China | CCBAU 05617 | HM057528 | | China |
| 15 | CCBAU 110 | EU155088 | China | LMG 17930 | AM295355 | | Belgium |
| 16 | HAMBI 2910 | GU994064 | China | HAMBI 2910 | GU994046 | | China |
| 17 | ORS1009 | AF169590 | UK | ORS1009 | DQ411967 | | Mexico |
| 18 | BR_922 | MN272276 | Brazil | CFNEI 156 | DQ411956 | | Mexico |
| 19 | HAMBI 1489 | GU994063 | China | HAMBI 1489 | DQ411960 | | Mexico |
| 20 | CCBAU 05557 | HM057491 | China | USDA 205 | DQ411957 | | Mexico |
| 21 | GS0466-2 | KJ606273 | China | USDA 1002 | DQ411964 | | Mexico |
