# Supplementary material for: Nickel mine soil is a potential source for soybean plant growth promoting and heavy metal tolerant rhizobia
Source: PeerJ. 2022 Apr 21;10:e13215. doi: 10.7717/peerj.13215 (PMC9035279; doi:10.7717/peerj.13215)
Supplement: Table S5 [file peerj-10-13215-s005.docx]

**Table S5.** Reference strains used in *nifH* phylogenetic tree.

| ***nifH*** | | | ***nifH*** | | |
| --- | --- | --- | --- | --- | --- |
| Strain | ID | Country | Strain | ID | Country |
| ORS1009 | Z95218 | Finland | UPM-Ca36 | DQ407296 | Spain |
| AC14c | JF450359 | Norway | WSM1271 | AY601521 | Australia |
| HAMBI 1489 | DQ411934 | Mexico | CCBAU 61158 | DQ311093 | China |
| CCBAU 65708 | EU622088 | China | RP254 | DQ413016 | UK |
| ORS609 | Z95221 | Finland | CCBAU 71714 | DQ411931 | Mexico |
| CCBAU 65798 | EU622087 | China | CCBAU 83325 | EF549533 | China |
| CCBAU 23314 | HQ231554 | China | CIAT 899 | JX863573 | Mexico |
| CCBAU110 | DQ411933 | Mexico | BLR175 | CP071615 | China |
| CSLC115N | NZPZJX 01000070 | Argentina | USDA 2370 | DQ450935 | Portugal |
| ATCC 11325 | AY945954 | Spain | OO99 | JN186287 | Canada |
| HBR78 | JN580785 | Finland |  |  |  |
